# Supplementary material for: 5-(N-Trifluoromethylcarboxy)aminouracil as a Potential DNA Radiosensitizer and Its Radiochemical Conversion into N-Uracil-5-yloxamic Acid
Source: Int J Mol Sci. 2020 Sep 1;21(17):6352. doi: 10.3390/ijms21176352 (PMC7504071; doi:10.3390/ijms21176352)
Supplement: Supplementary file 1 [file ijms-21-06352-s001.pdf]

Supplementary Materials

5-(*N*-Trifluoromethylcarboxy)aminouracil as a Potential DNA Radiosensitizer and its Radiochemical Conversion into *N*-uracil-5-yloxamic Acid

Paulina Spisz, Witold Kozak, Lidia Chomicz-Mańka, Samanta Makurat, Karina Falkiewicz, Artur Sikorski, Anna Czaja, Janusz Rak, Magdalena Zdrowowicz*

Department of Physical Chemistry, Faculty of Chemistry, University of Gdańsk, Wita Stwosza 63, 80-308 Gdańsk, Poland; paulina.rewers@phdstud.ug.edu.pl (P.S.); davelombardo@wp.pl (W.K.); lidia.chomicz-manka@ug.edu.pl (L.C-M.) samanta.makurat@ug.edu.pl (S.M.); karina.falki@gmail.com, (K.F.); artur.sikorski@ug.edu.pl (A.S.); czaja.ania@yahoo.com (A.C.); janusz.rak@ug.edu.pl (J.R.);

* Correspondence: magdalena.zdrowowicz@ug.edu.pl; (M.Z.)

Received: date; Accepted: date; Published: date

Table of Content

NMR spectra of 5-(*N*-trifluoromethylcarboxy)aminouracil (Figure S1-S2) S3

MS and MS/MS spectra of 5-(*N*-trifluoromethylcarboxy)aminouracil (Figure S3-S4) S4

MS and MS/MS spectra of a radioproduct (Figure S5-S6) S5

Crystallographic data for 5-(*N*-trifluoromethylcarboxy)aminouracil (Table S1-S2) S6

Plating efficiencies and survival fractions (clonogenic assay) (Table S3) S8

Hypothetic pathway for the formation of 2-oxazolidinone ring (Figure S7) S9

Kinetic model (Scheme S1, Equation S1, Table S4) S10

Cytometric analysis of histone H2A.X phosphorylation (Figure S8) S13

Cytotoxicity assay (Figures S9 and S10) S14

References S15

NMR spectra of 5-(*N*-trifluoromethylcarboxy)aminouracil

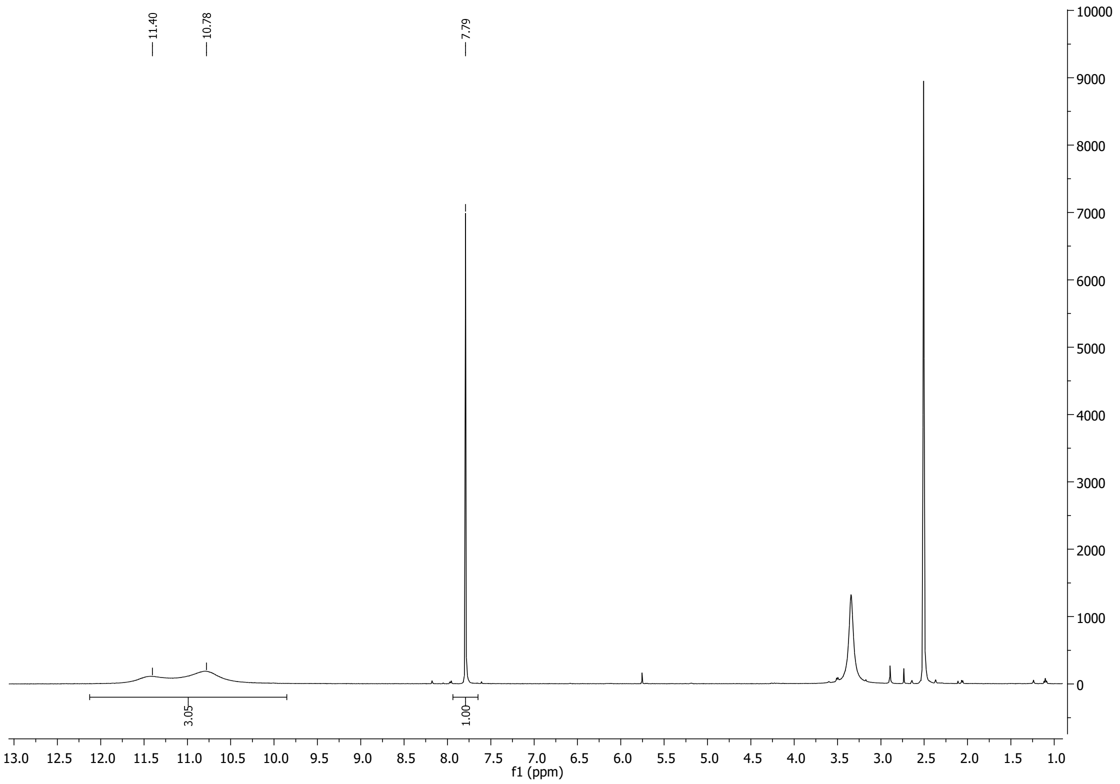

Figure S1. ^{1}H NMR spectrum of 5-(*N*-trifluoromethylcarboxy)aminouracil.

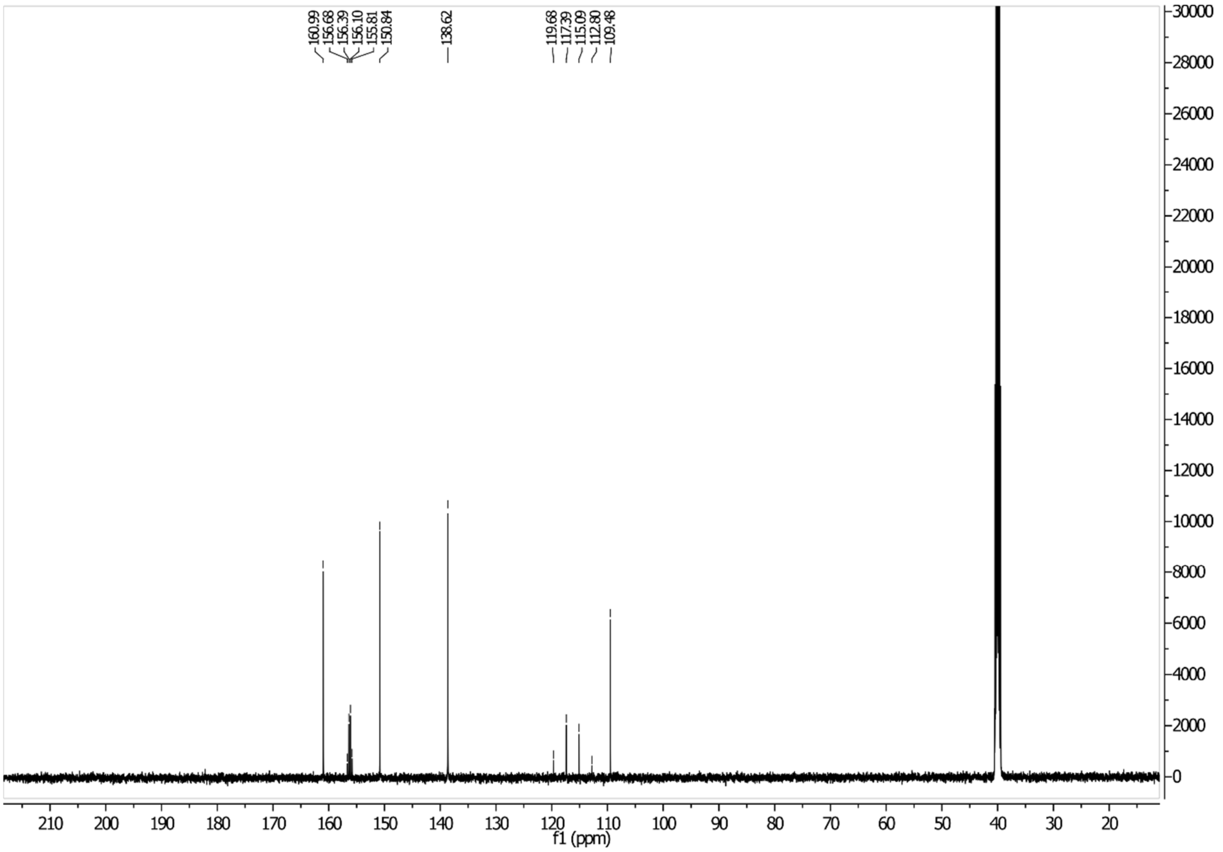

Figure S2. ^{13}C NMR spectrum of 5-(*N*-trifluoromethylcarboxy)aminouracil.

MS and MS/MS spectra of a 5-(*N*-trifluoromethylcarboxy)aminouracil

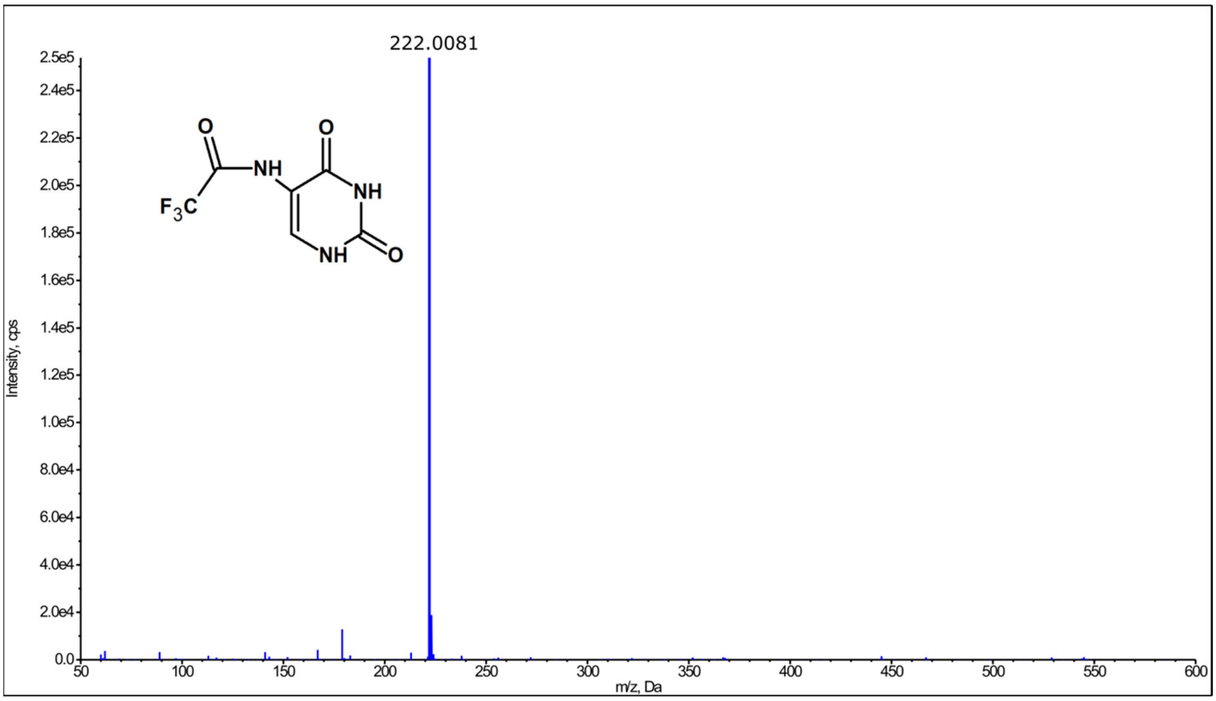

Figure S3. The MS spectrum (in negative ionization mode) of 5-(*N*-trifluoromethylcarboxy)-aminouracil.

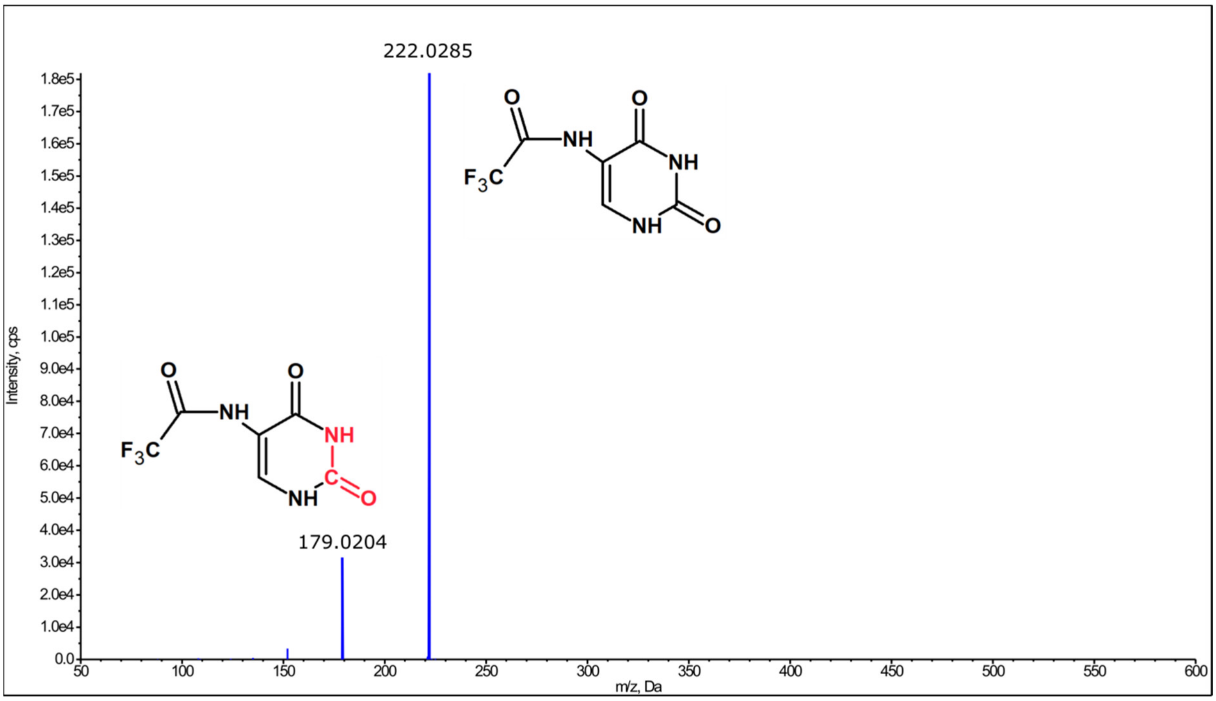

Figure S4. The MS/MS spectrum (in negative ionization mode) of 5-(*N*-trifluoromethyl-carboxy)aminouracil and ion identities.

MS and MS/MS spectra of a radioproduct

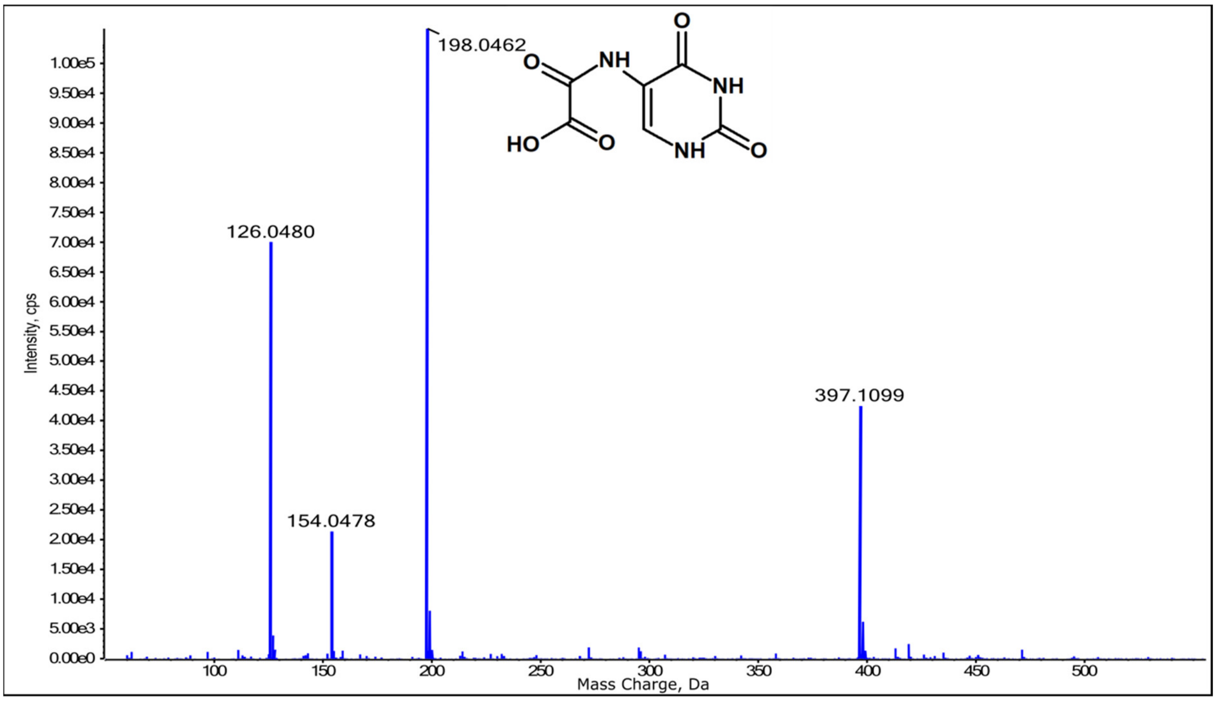

Figure S5. The MS spectrum (in negative ionization mode) of radioproduct.

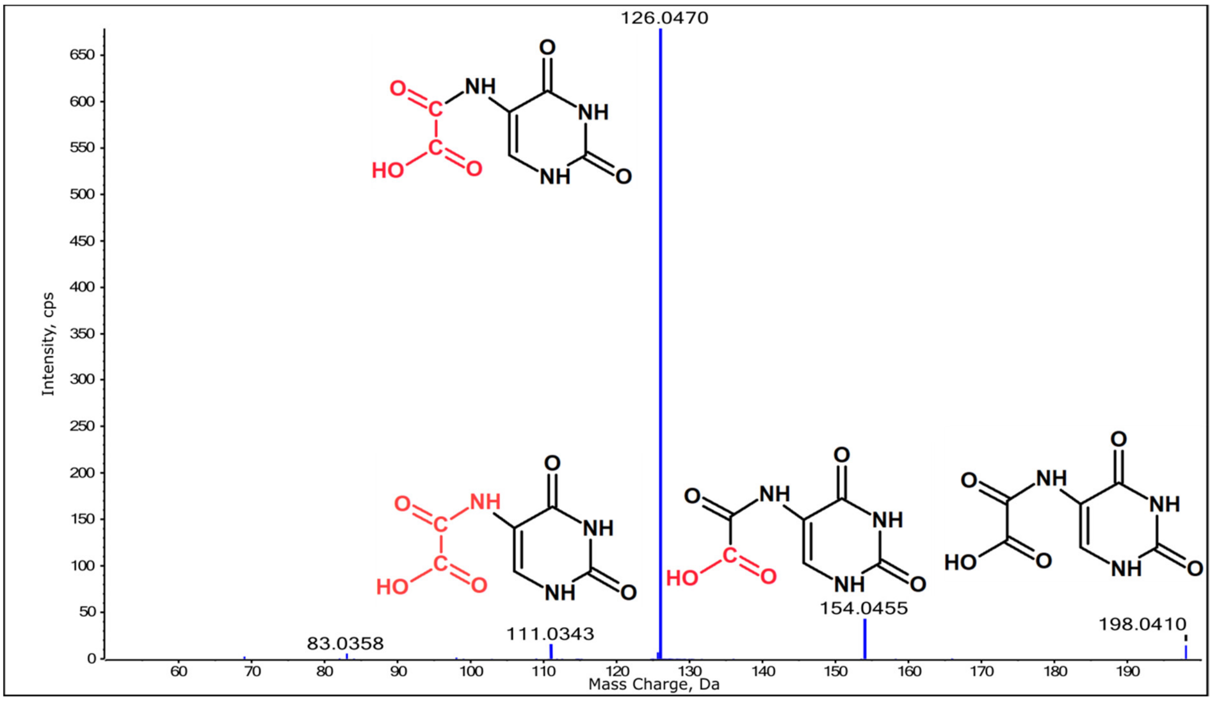

Figure S6. The MS/MS spectrum (in negative ionization mode) of radioproduct and ion identities.

Crystallographic data for 5-(*N*-trifluoromethylcarboxy)aminouracil

Table S1. Crystal data and structure refinement parameters for 5-(*N*-trifluoromethylcarboxy)aminouracil.

Chemical formula	$C_6H_4N_3F_3O_3$
FW/g · mol^{-1}	223.12
Crystal system	monoclinic
Space group	C2/c
a/Å	19.616(3)
b/Å	7.268(2)
c/Å	11.663(3)
α/°	90
β/°	100.55(2)
γ/°	90
V/Å^3	1634.8(6)
Z	8

T/K	295(2)
λ_{Mo}/Å	0.71073
ρ_{calc}/g·cm^{-3}	1.813
F(000)	896
μ/mm^{-1}	0.187
θ range/°	3.38–25.00
Completness θ/%	99.9
Reflections collected	5089
Reflections unique	1441 [R_{int} = 0.1635]
Data/restraints/parameters	1441/0/145
Goodness of fit on F^2	0.957
Final R_1 value (I>2σ(I))	0.0706
Final wR_2 value (I>2σ(I))	0.1259
Final R_1 value (all data)	0.1938
Final wR_2 value (all data)	0.1733
CCDC number	2016475

Table S2. Hydrogen bonding interactions in the crystal structure of the title compound.

D–H···A	d(D–H) (Å)	d(H···A) (Å)	d(D···A) (Å)	<D–H···A (°)
N1–H1···O7^{i}	0.99(6)	1.83(6)	2.821(6)	174(4)
N3–H3···O8^{ii}	0.77(6)	2.13(6)	2.878(6)	165(6)
N9–H9···O8^{iii}	0.86(6)	2.04(6)	2.823(6)	151(5)
C6–H6···O11^{iv}	0.93	2.58	3.284(7)	133

Symmetry codes:(i) 1/2−x,−1/2−y,1−z; (ii) 1−x,−y,1−z; (iii) 1−x,y,3/2−z; (iv) 1/2−x,−1/2+y,3/2−z.

Plating efficiencies (clonogenic assay)

Table S3. Plating efficiencies and survival fractions (obtained from clonogenic assay) for the PC3 cells treated with 5-(*N*-trifluoromethylcarboxy)aminouracil and/or radiation.

Dose [Gy]	0 µM CF_3CONHU		100 µM CF_3CONHU	
	Plating efficiency	Survival fraction	Plating efficiency	Survival fraction
0	40.66 ± 0.09	100.0	42.09 ± 0.22	100.0
0.5	35.06 ± 0.56	86.2 ± 2.2	28.88 ± 2.25	68.6 ± 7.1
1	28.25 ± 1.25	69.5 ± 4.1	23.81 ± 0.44	56.6 ± 1.9
2	17.63 ± 0.75	43.4 ± 2.8	13.68 ± 1.31	32.5 ± 4.6
4	4.69 ± 0.94	11.5 ± 3.2	1.75 ± 0.38	4.2 ± 1.3

Hypothetic pathway for the formation of 2-oxazolidinone ring

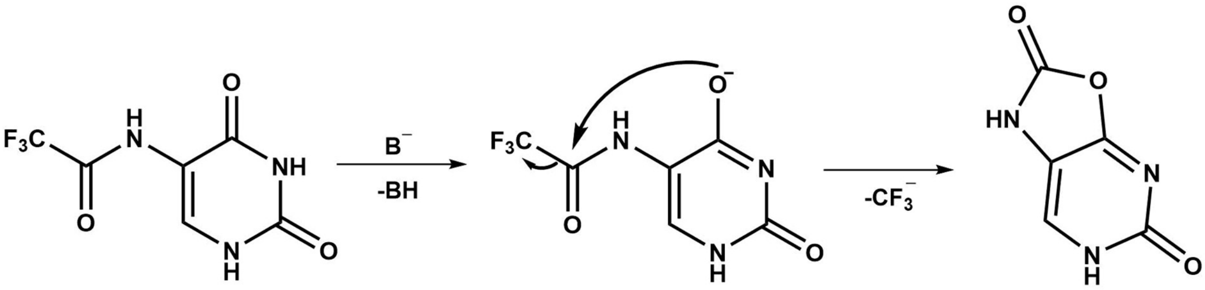

Figure S7. Hypothetic pathway for the formation of 2-oxazolidinone ring.

Kinetic model

In order to mimic the experimental conditions k_1 (Scheme S1) was assumed to be equal to $3.3 \cdot 10^{-8}$, which corresponds to the concentration of solvated electrons and hydroxyl radicals generated by the experimental dose rate (0.117 Gy $\cdot$ s^{-1}). We further assumed that the concentration of water, *t*-butanol (both used in large excess) and hydroxyl anions (the sample was buffered, see point 2.2.3 Radiolysis) was constant during the experiment and equal to 55.5, $3.0 \cdot 10^{-2}$ and $1.0 \cdot 10^{-7}$ M, respectively. For k_2 (Scheme S1), the rate reported for the reaction between e_{hyd} and pyrimidine was assumed [S1], while for k_3 (Scheme S1) the value assigned for the reaction rate between the $^{\bullet}OH$ radicals and *t*-butanol, while k_6 results from the Debye equation for water and T = 298 K [S2]. Finally k_{14} (Scheme S1) was assumed to be equal to the rate evaluated by Mezyk and Madden [S3] for the self –recombination of *t*-butyl alcohol radicals in water. The remaining rate constants were obtained using transition state theory and ΔG^* calculated at the M06-2X/6-31++G(d,p) level (Figure 6). The system of differential equations (Equation S1) matching the mechanism depicted in Scheme 1 was integrated for 1200 s and then for further 60 000 s with k_1 (Scheme S1) set to 0, that corresponded to X-ray source turned off. Reactions 1, 2, 3 and 9 (Scheme S1) were assumed to be irreversible since the thermodynamic stimuli for the reverse processes were highly unfavorable (from 17.4 for reaction (3) to even 67.2 kcal $\cdot$ mol^{-1} for reaction (9)) making the reverse reactions completely improbable at the ambient temperature.

$$\mathrm{H_2O} \xrightarrow{X,\mathrm{k_1}} \mathrm{OH^{\bullet}} + e_{sol} \tag{1}$$

$$[\mathrm{CF_3-CO-R}] + e_{sol} \xrightarrow{\mathrm{k_2}} [\mathrm{CF_3-CO-R}]^{\bullet-} \tag{2}$$

$$[t-\mathrm{But}] + [\mathrm{OH}]^{\bullet} \xrightarrow{\mathrm{k_3}} [t-\mathrm{But}]^{\bullet} + \mathrm{H_2O} \tag{3}$$

$$[\mathrm{CF_3-CO-R}]^{\bullet-} \underset{\mathrm{k_5}}{\overset{\mathrm{k_4}}{\rightleftarrows}} [\mathrm{CF_2-CO-R}]^{\bullet} + \mathrm{F^-} \tag{4}$$

$$[\mathrm{CF_2-CO-R}]^{\bullet} + \mathrm{OH^-} \underset{\mathrm{k_7}}{\overset{\mathrm{k_6}}{\rightleftarrows}} [\mathrm{CF_2OH-CO-R}]^{\bullet-} \tag{5}$$

$$[\mathrm{CF_2OH-CO-R}]^{\bullet-} \underset{\mathrm{k_9}}{\overset{\mathrm{k_8}}{\rightleftarrows}} [\mathrm{CFOH-CO-R}]^{\bullet} + \mathrm{F^-} \tag{6}$$

$$[\mathrm{CFOH-CO-R}]^{\bullet} + \mathrm{H_2O} \underset{\mathrm{k_{11}}}{\overset{\mathrm{k_{10}}}{\rightleftarrows}} [\mathrm{CO-CO-R}]^{\bullet} + \mathrm{H_2O} + \mathrm{HF} \tag{7}$$

$$[\mathrm{CO-CO-R}]^{\bullet} + \mathrm{H_2O} \underset{\mathrm{k_{13}}}{\overset{\mathrm{k_{12}}}{\rightleftarrows}} [\mathrm{COOH-COH-R}]^{\bullet} \tag{8}$$

$$[\mathrm{COOH-COH-R}]^{\bullet} + [t-\mathrm{But}]^{\bullet} \xrightarrow{\mathrm{k_{14}}} [\mathrm{COOH-CO-R}] + [t-\mathrm{But}] \tag{9}$$

Scheme S1. Elementary reactions leading from the radical anion of CF_3CONHU to *N*-uracil-5-yloxamic acid. R = NHU.

Equation S1. System of kinetic equations used for predicting the time of reaction completion.

$$1)\ \frac{d[e_{sol}]}{dt} = k_1 - k_2[CF_3 - CO - R][e_{sol}]$$

$$2)\ \frac{d[OH]^\bullet}{dt} = k_1 - k_3[OH]^\bullet[t - But]$$

$$3)\ \frac{d[CF_3 - CO - R]^{\bullet-}}{dt} = -k_4[CF_3 - CO - R]^{\bullet-} + k_5[CF_2 - CO - R]^\bullet[F^-] + k_2[CF_3 - CO - R][e_{sol}]$$

$$4)\ \frac{d[CF_2 - CO - R]^\bullet}{dt} = -k_6[CF_2 - CO - R]^\bullet[OH^-] + k_7[CF_2OH - CO - R]^{\bullet-} - k_5[CF_2 - CO - R]^\bullet[F^-] + k_4[CF_3 - CO - R]^{\bullet-}$$

$$5)\ \frac{d\,[CF_2OH - CO - R]^{\bullet-}}{dt} = -k_8\,[CF_2OH - CO - R]^{\bullet-} + k_9\,[CFOH - CO - R]^\bullet\,[F^-] - k_7[CF_2OH - CO - R]^{\bullet-} + k_6[CF_2 - CO - R]^\bullet[OH^-]$$

$$6)\ \frac{d[CFOH - CO - R]^\bullet}{dt} = -k_{10}[CFOH - CO - R]^\bullet[H_2O] + k_{11}[CO - CO - R]^\bullet[H_2O][HF] - k_9[CFOH - CO - R]^\bullet[F^-] + k_8[CF_2OH - CO - R]^{\bullet-}$$

$$7)\ \frac{d[F^-]}{dt} = k_4[CF_3 - CO - R]^{\bullet-} - k_5[CF_2 - CO - R]^\bullet[F^-] + k_8\,[CF_2OH - CO - R]^{\bullet-} - k_9\,[CFOH - CO - R]^\bullet\,[F^-]$$

$$8)\ \frac{d[CO - CO - R]^\bullet}{dt} = -k_{12}[CO - CO - R]^\bullet[H_2O] + k_{13}[COOH - COH - R]^\bullet - k_{11}[CO - CO - R]^\bullet[H_2O][HF] + k_{10}[CFOH - CO - R]^\bullet[H_2O]$$

$$9)\ \frac{d[HF]}{dt} = k_{10}[CFOH - CO - R]^\bullet[H_2O] - k_{11}[CO - CO - R]^\bullet[H_2O][HF]$$

$$10)\ \frac{d\,[COOH - COH - R]^\bullet}{dt} = -k_{13}\,[COOH - COH - R]^\bullet + k_{12}[CO - CO - R]^\bullet[H_2O] - k_{14}[COOH - COH - R]^\bullet[t - But]^\bullet$$

$$11)\ \frac{d\,[COOH - CO - R]}{dt} = k_{14}\,[COOH - COH - R]^\bullet[t - But]^\bullet$$

$$12)\ \frac{d\,[CF_3 - COR]}{dt} = -k_2\,[CF_3 - CO - R][e_{sol}]$$

$$13)\ \frac{d\,[t - But]^\bullet}{dt} = k_3[t - But][OH]^\bullet - k_{14}\,[COOH - COH - R]^\bullet[t - But]^\bullet$$

Table S4. Rate constants (T = 298 K) employed in the kinetic model shown in Scheme S1. The values of particular constants were obtained using transition state theory and activation free energies (ΔG^*) calculated at the M06-2X/6-31++G(d,p) level. [^-OH], [H_2O] and [*t*-butanol] equal to 10^{-7}, 55.5 and $3 \cdot 10^{-2}$ M, respectively.

Constant (**Scheme S1**)	kT/h*exp(-ΔG*/(RT))	Invariant concentration species	Rate constant used in the kinetic calculations (**Equation S1**)
k_1	$3.3 \cdot 10^{-8}$ [M s^{-1}]		$3.3 \cdot 10^{-8}$ [M s^{-1}]
k_2 [S1]	$2.0 \cdot 10^{10}$ [M^{-1} s^{-1}]		$2.0 \cdot 10^{10}$ [M^{-1} s^{-1}]
k_3 [S4]	$6.0 \cdot 10^{8}$ [M^{-1} s^{-1}]		$6.0 \cdot 10^{8}$ [M^{-1} s^{-1}]
k_4	$5.07 \cdot 10^{5}$ [s^{-1}]		$5.07 \cdot 10^{5}$ [s^{-1}]
k_5	$3.36 \cdot 10^{12}$ [M^{-1} s^{-1}]		$3.36 \cdot 10^{12}$ [M^{-1} s^{-1}]
k_6 [S2]	$7.4 \cdot 10^{9}$ [M^{-1} s^{-1}]	^-OH	$7.4 \cdot 10^{2}$ [s^{-1}]
k_7	$1.23 \cdot 10^{-18}$ [s^{-1}]		$1.23 \cdot 10^{-18}$ [s^{-1}]
k_8	$9.38 \cdot 10^{4}$ [s^{-1}]		$9.38 \cdot 10^{4}$ [s^{-1}]
k_9	$1.20 \cdot 10^{9}$ [M^{-1} s^{-1}]		$1.20 \cdot 10^{9}$ [s^{-1}]
k_{10}	$5.99 \cdot 10^{6}$ [M^{-3} s^{-1}]	H_2O	$3.27 \cdot 10^{-4}$ [M^{-2} s^{-1}]
k_{11}	$1.03 \cdot 10^{1}$ [M^{-2} s^{-1}]	H_2O	$5.72 \cdot 10^{2}$ [M^{-1} s^{-1}]
k_{12}	$1.84 \cdot 10^{5}$ [M^{-1} s^{-1}]	H_2O	$1.02 \cdot 10^{7}$ [s^{-1}]
k_{13}	$1.53 \cdot 10^{-9}$ [s^{-1}]		$1.53 \cdot 10^{-9}$ [s^{-1}]
k_{14} [S3]	$1.20 \cdot 10^{9}$ [M^{-1} s^{-1}]	*t*-butanol	$3.6 \cdot 10^{7}$ [M^{-1} s^{-1}]

Cytometric analysis of histone H2A.X phosphorylation

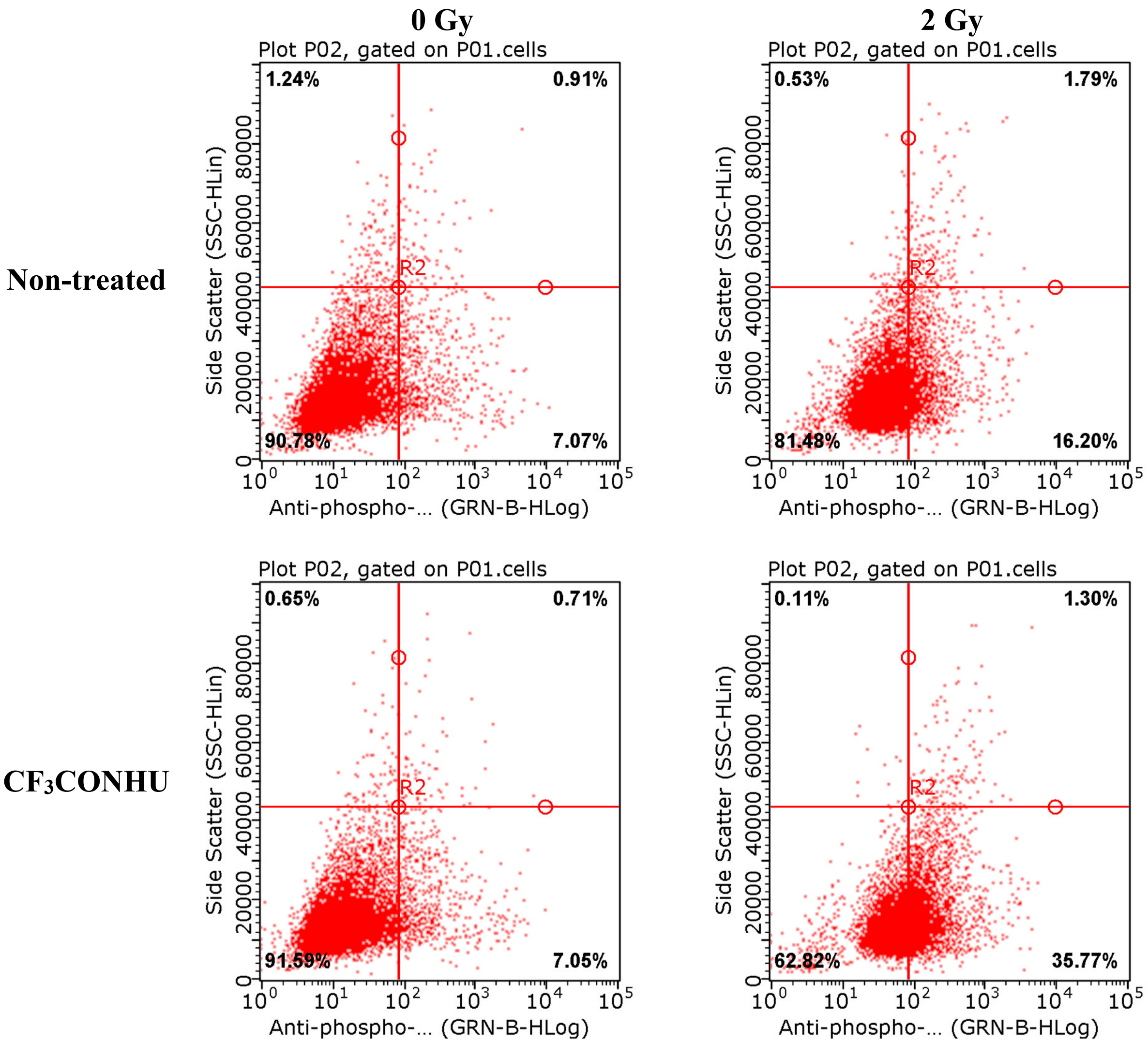

Figure S8. Flow cytometric analysis of H2A.X phosphorylation. γH2A.X was measured 1 h after irradiation.

Cytotoxicity assay

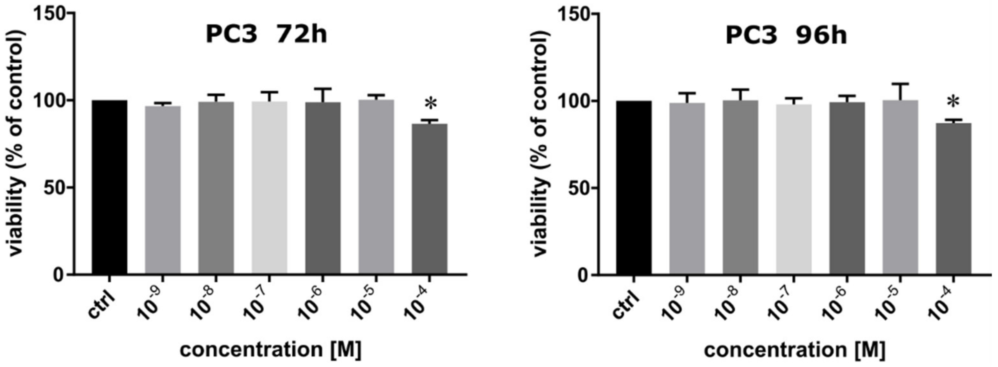

Figure S9. The viability of PC3 cells after 72 and 96 h treatment with 5-(*N*-trifluoromethylcarboxy)aminouracil in a range of concentrations from 0 to 10^{-4} M. Results are shown as mean ± SD of three independent experiments performed in triplicate.

*statistically significant difference is present between treated culture compared with control (untreated culture)

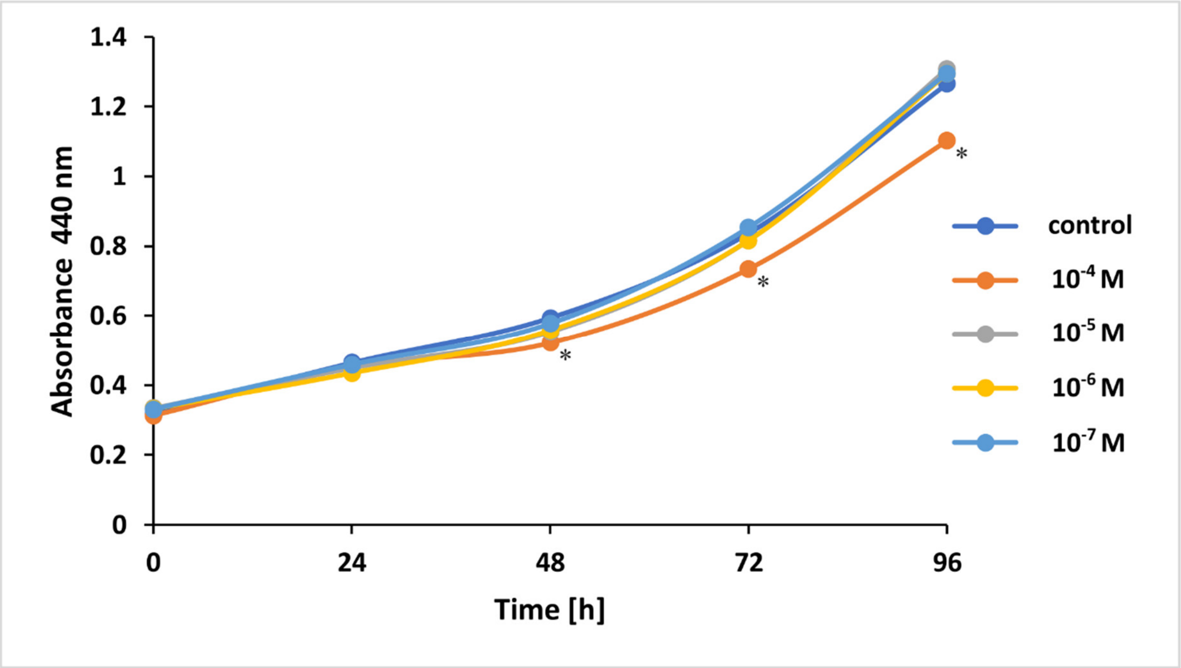

Figure S10. Cell proliferation measured using WST-1 assay. The absorbance at 440 nm plotted as a function of time.

*statistically significant difference is present between treated culture compared with control (untreated culture)

References

S1. Steenken, S. Purine bases, nucleosides, and nucleotides: aqueous solution redox chemistry and transformation reactions of their radical cations and e- and OH adducts. *Chem. Rev.* **1989**, *89*, 503–520.
S2. Debye, P. Reaction rates in ionic solutions. *Trans. Electrochem. Soc.* **1942**, *82*, 265–272.
S3. Mezyk S.P.; Madden, K.P. Self-recombination rate constants for 2-propanol and tert-butyl alcohol radicals in water. *J. Phys. Chem. A* **1999**, *103*, 235–242.
S4. Buxton, G.V.; Greenstock, C.L.; Helman, W.P.; Ross, A.B. Critical review of rate constants for reactions of hydrated electrons, hydrogen atoms and hydroxyl radicals ($^{\bullet}OH/^{\bullet}O^{-}$) in aqueous solution. *J. Phys. Chem. Ref. Data* **1988**, *17*, 513–886.
